# Supplementary material for: Placental Remote Control of Fetal Metabolism: Trophoblast mTOR Signaling Regulates Liver IGFBP-1 Phosphorylation and IGF-1 Bioavailability
Source: Int J Mol Sci. 2023 Apr 14;24(8):7273. doi: 10.3390/ijms24087273 (PMC10138459; doi:10.3390/ijms24087273)
Supplement: Supplementary file 1 [file ijms-24-07273-s001.zip › ijms-2267382-supplementary.pdf]

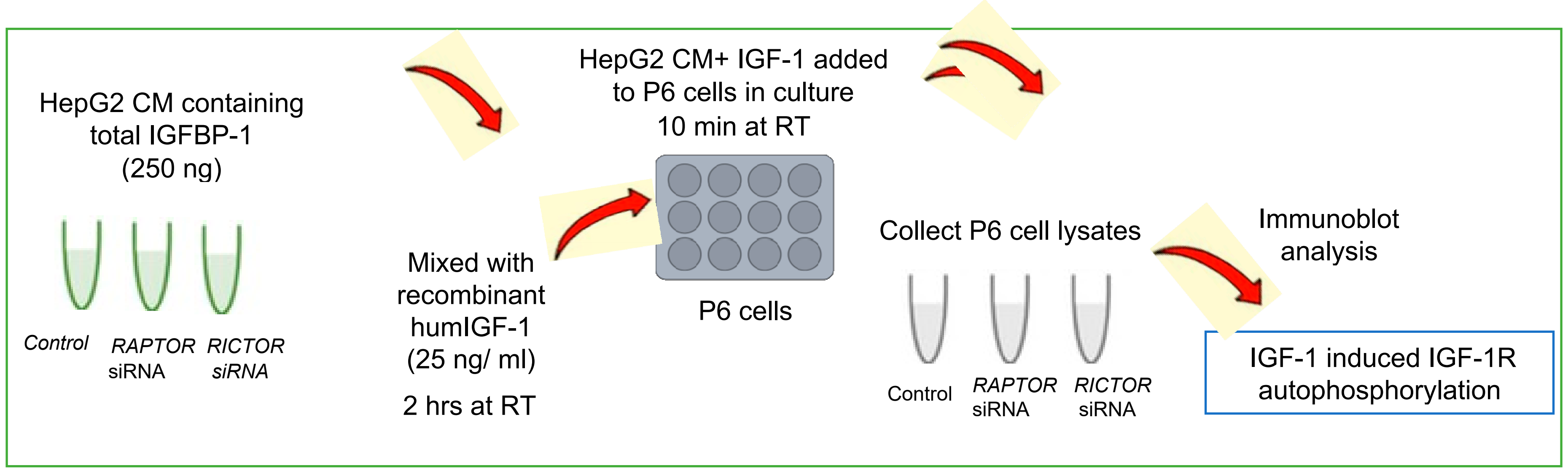

Supplemental Figure S1: Schematic representation of the experimental model (P6 (BALB/c3T3) cells overexpress human IGF-1R but do not express IGF-1) to examine whether the alterations in IGFBP-1 phosphorylation functionally alter IGF-1 receptor (IGF-1R) autophosphorylation. Conditioned media from HepG2 cells from respective PHT cell treatments were incubated with rhIGF-1 (25 ng/mL) for two hours at room temperature. IGF-1 (10 ng) served as a positive control. P6 cells were then treated for 10 minutes with the P6 media containing IGFBP-1: IGF-I complexes or with IGF-1 alone (positive control] and were used to treat serum-starved P6 cells (75% confluent) in culture dishes for 10 min. The P6 cells were collected to assess IGF-1R autophosphorylation using phospho-site specific IGF-1Rβ (Tyr1135) primary antibody.
